# Molecular insights into the bidirectional link between anxiety and COVID-19: a combined clinical and bioinformatics approach

**DOI:** 10.3389/fpsyt.2025.1643355

**Published:** 2025-07-22

**Authors:** Wenjie Huang, Biao Hu, Chengyu Gu, Hao Wu, Yize Huang, Dexiang Yang

**Affiliations:** ^1^ Department of Pulmonary and Critical Care Medicine, Tongling Hospital Affiliated to Bengbu Medical University, Tongling, Anhui, China; ^2^ Department of Psychiatry, Tongde Hospital of Zhejiang Province, Hangzhou, Zhejiang, China; ^3^ Department of Infectious Diseases, Tongling People’s Hospital Affiliated to Bengbu Medical University, Tongling, Anhui, China

**Keywords:** COVID-19, anxiety, signal pathway, gene expression, SAS

## Abstract

**Introduction:**

Numerous studies have reported an increased incidence of anxiety in individuals affected by COVID-19; however, the specific molecular mechanisms underlying this association remain poorly understood.

**Methods:**

In this study, we employed the Zung Self-Rating Anxiety Scale (SAS) to assess anxiety levels in 36 asymptomatic COVID-19 patients. In parallel, we conducted a comprehensive literature-based data mining analysis to reconstruct the functional and molecular pathways linking COVID-19 and anxiety. Additionally, we performed a meta-analysis using eight independent COVID-19 case–control gene expression datasets to examine expression alterations in the literature-derived pathways.

**Results:**

Our findings revealed that even among asymptomatic individuals, approximately 25% exhibited mild anxiety symptoms, which negatively correlated with age. The reconstructed pathways suggested that COVID-19 may contribute to cognitive decline through multisystem dysfunction and structural or functional brain abnormalities—hallmarks of anxiety disorders. The meta-analysis confirmed increased expression of four anxiety-related molecular mediators in response to COVID-19 infection: CALCA, TNF, PLAT, and PPARG, with the latter three associated with neurocognitive decline.

**Conclusion:**

These results provide molecular-level evidence for a bidirectional association between COVID-19 and anxiety, potentially mediated by dysregulated inflammatory cytokines and other secreted proteins. Furthermore, impaired cognitive function may serve as a critical link connecting these two conditions.

## 1 Introduction

Anxiety disorders are among the most common psychiatric conditions globally, affecting over 300 million people in 2019 (1). They are linked to impaired functioning, higher risk of chronic disease, and reduced quality of life (2, 3), yet remain underdiagnosed and undertreated, especially in underserved populations (4).

Severe acute respiratory syndrome coronavirus 2 (SARS-CoV-2) causes COVID-19, with an infection fatality rate (IFR) of 0.5% to 2%, increasing to 4.6% at age 75 and 15% at age 85 (5, 6). It infects multiple body systems, including the respiratory tract (7). Older COVID-19 patients may experience brain tissue loss (0.2%–2%) in regions linked to smell, taste, and cognition (8). Beyond respiratory symptoms, COVID-19 is associated with neuropsychiatric effects; retrospective studies show increased anxiety incidence post-infection, with hazard ratios near 1.3 compared to other respiratory infections (9). These outcomes may result from neuroinflammation and direct viral CNS effects, though it remains unclear if the damage is reversible (7, 10). However, it is not currently known whether this damage is reversible or permanent.

Studies have reported increased depression and anxiety symptoms during the COVID-19 pandemic (11, 12). One survey found 25% of COVID-19 patients had anxiety symptoms (13), and over 9% expressed negative self/world views (14). Google Trends also showed heightened public concern about anxiety (15). COVID-19 has been linked to structural changes in brain regions involved in anxiety, such as the hippocampal gyrus and orbital cortex (16–18), as well as damage to the immune, nervous, and microvascular systems (19), all of which show anxiety-related pathology (20–24). These effects are also connected to cognition, a core feature of anxiety (25). Most existing studies are symptom-based, highlighting the need for molecular-level investigations.

Anxiety may influence COVID-19 both directly and indirectly. It can impair immune function by elevating stress hormones like cortisol, increasing susceptibility to infection (26). Indirectly, anxiety affects behaviors related to the pandemic, leading to actions such as stockpiling, noncompliance with health guidelines, and panic-driven decisions (27–29). These responses, along with links between COVID-related anxiety and perceived health risks (30), highlight the importance of integrating mental health support into pandemic response strategies.

This study aims to uncover molecular links between COVID-19 and anxiety to better understand their shared symptoms and high comorbidity. We first analyzed Zung Self-Rating Anxiety Scale (SAS) data from 36 asymptomatic COVID-19 patients. We then conducted a large-scale literature review to map functional and molecular pathways connecting the two conditions. A meta-analysis (31) was also performed to assess gene expression changes in COVID-19 patients. Our results indicate that COVID-19 can activate anxiety-related molecules, while anxiety may in turn exacerbate COVID-19 through molecular mechanisms.

## 2 Method

### 2.1 Zung Self-Rating Anxiety Scale

From April 4th to April 8th, 2022, a psychological evaluation was conducted in the isolation ward of the Infectious Diseases Department of Tongling People’s Hospital, which is a designated treatment center for patients with novel coronavirus infection in Anhui Province’s Tongling City. There were total 36 participants completed an online questionnaire using smartphones and the online Zung Self-Rating Anxiety Scale (SAS) (32, 33). The SAS has shown good reliability with an internal consistency reliability coefficient of 0.80 (34).

A Pearson correlation analysis was conducted between the SAS total score and the age and gender. The unit for the age is by year; the male was numbered as 1, and the female as 2. The raw SAS total score can be converted to an ‘Anxiety Index’ score (AIS) by multiplying the raw score by 1.25. Subjects are classified into four groups according to their AIS: normal (<45), mild depression (45 to 59), moderate to marked major depression (60 to 74), and severe to extreme major depression (≥75) (35). The Ethics Committee of the People’s Hospital of Tongling has approved this study. A written consent form was obtained from each included patient for using the datasets for publication. To note, while the AIS derived from the Zung SAS was used to define anxiety groups in this study, no cross-validation with other anxiety screening tools (e.g., the Hospital Anxiety and Depression Scale [HADS]) was conducted. Given the constraints of data collection during isolation, the Zung SAS was selected for its simplicity, reliability, and suitability for remote administration. Nonetheless, future studies should consider including multiple validated instruments to allow for cross-method comparisons and improved diagnostic precision.

Specifically, we used the sampsizepwr() function in MATLAB (R2020a) to estimate the minimum required sample size to detect differences in Anxiety Index Scores (AIS) across standard thresholds (45, 60, and 74) with 95% statistical power. The function syntax is sampsizepwr(‘t2’, [μ_1_ σ], μ_2_, power), where μ_1_ and μ_2_ are group means, and σ is the standard deviation. We initially used σ = 10 based on prior literature (35) for theoretical illustration:

We used the sampsizepwr() function in MATLAB (R2020a) to estimate the minimum required sample size to detect differences in Anxiety Index Scores (AIS) across standard thresholds (45, 60, and 74) with 95% statistical power. The function syntax is sampsizepwr(‘t2’, [μ_1_ σ], μ_2_, power), where μ_1_ and μ_2_ are group means, and σ is the standard deviation. We initially used σ = 10 based on prior literature (35) for theoretical illustration.

To evaluate separation across all cutoff combinations:

sampsizepwr(‘t2’, [45 10], 60, 0.95) → 13 participants per group.
sampsizepwr(‘t2’, [45 10], 74, 0.95) → 5 participants per group.
sampsizepwr(‘t2’, [60 10], 74, 0.95) → 15 participants per group.

However, for our actual dataset, the group parameters are:

Milder anxiety group (g1): n_1_ = 9, mean_1_ = 48.61, std_1_ = 5.12.
Control group (g2): n_2_ = 27, mean_2_ = 36.30, std_2_ = 3.82.

We calculated the pooled standard deviation as:


$$s\_pooled=sqrt((std_{1}^{2}+std_{2}^{2})/2)=4.85$$

Using our real data, the power analysis yields:

sampsizepwr(‘t2’, [48.61 4.85], 36.30, 0.95) → 6 participants per group.

Thus, our actual sample sizes (n_1_ = 9, n_2_ = 27) exceed this threshold and are adequate to detect statistically significant differences in AIS with 95% power. We emphasize that this refers to the power to detect differences in group means, not to diagnostic classification accuracy.

The assumption of normality was based on findings from Dunstan and Scott (36), who reported that Zung SAS scores in general population samples approximate a normal distribution. While this supports the rationale for applying a parametric method such as sampsizepwr(), we acknowledge that the distributional characteristics of AIS in our specific clinical sample were not empirically tested. Therefore, this power analysis serves as a basic check on sample adequacy for detecting threshold-based group differences and does not constitute psychometric validation. This limitation is discussed in detail in the Discussion section, and we recommend that future studies validate these assumptions in larger, diverse cohorts.

The study focused on asymptomatic patients with COVID-19 who were infected with the Omicron BA.2 variant and admitted to the first and second wards of the isolation ward in the Infectious Diseases Department of Tongling People’s Hospital. These patients tested positive for SARS-CoV-2 in respiratory and other specimens, but did not exhibit clinical symptoms such as fever, cough, or sore throat (37). To be included in the study, patients had to meet the following criteria: 1) asymptomatic COVID-19 infection; 2) normal communication and clear consciousness; 3) voluntary participation and informed consent. The asymptomatic infection has been defined as follows: (1) positive results from two nasal and/or throat swabs tested by reverse transcription polymerase chain reaction (RT-PCR) within 24 hours; (2) absence of self-perceived clinical symptoms such as fever, cough, fatigue, sore throat, and muscle aches; (3) positive for SARS-CoV-2 etiological test in respiratory specimens, with or without detectable pulmonary imaging changes on chest computed tomography (CT). As prior research has established an increased sense of anxiety linked to COVID-19 (11, 12), our current investigation was carried out to evaluate how COVID-19 affects individuals who show no symptoms. Asymptomatic patients who did not meet the AIS > 45 cutoff served as an internal control group, allowing us to compare ‘infected with anxiety’ vs. ‘infected without anxiety’ without introducing external healthy cohorts. This study aims to shed more light on the notion that even those without symptoms, but impacted by COVID-19, remain at a heightened risk of encountering elevated levels of anxiety.

Patients with a history of mental illness, inability to communicate due to disturbed consciousness, or under 14 years old were excluded from the study. The hospital ethics committee approved this study.

Although our *a priori* power analysis (sampsizepwr, power = 0.95) indicated that 36 participants were sufficient to distinguish among AIS thresholds (45, 60, 74), this modest sample size falls below typical standards for psychometric validation of multi-item scales (e.g., N ≥ 300 for factor analysis) and may limit the generalizability of our findings (38, 39). We have noted this limitation in the Discussion and recommend that future studies use larger cohorts.

To note, the Zung SAS data serve as an initial clinical observation that COVID-19 infection may be associated with elevated anxiety, consistent with prior reports (11, 12). To explore possible biological mechanisms underlying this association, the study integrates literature-based pathway reconstruction and transcriptomic meta-analysis. These molecular analyses are designed to propose mechanistic explanations for the observed clinical anxiety symptoms. While this integrative framework bridges clinical and molecular perspectives, further experimental validation—such as proteomic, immunological, or cellular studies—is needed to confirm the pathways proposed. While this integrative framework bridges clinical and molecular perspectives, further experimental validation—such as proteomic, immunological, or cellular studies—is needed to confirm the pathways proposed.

### 2.2 Reconstruct COVID-19 driven pathways influencing anxiety

A comprehensive literature-based pathway reconstruction was conducted in May 2023 to explore the bidirectional molecular relationship between COVID-19 and anxiety. Multiple tools and resources were utilized, including Pathway Studio (http://www.pathwaystudio.com/, no longer available), the Entrez API (https://www.ncbi.nlm.nih.gov/books/NBK25501), and Google Scholar (https://scholar.google.com), to identify both downstream targets and upstream regulators of COVID-19 and anxiety. For automated data mining, a Python 3.10–based toolkit was developed using the Entrez module from the Bio package to retrieve relevant PubMed records. Google Scholar was employed to manually verify key findings and address coverage gaps. Pathway Studio served as the primary mining platform due to its NLP-based integration of curated relationships from the biomedical literature.

Search terms included “COVID-19,” “SARS-CoV-2,” and all known COVID-19-related genes, as well as “anxiety,” “psychological stress,” and all anxiety-related genes. Additional terms included tissue and organ systems implicated in both conditions, such as the immune system, nervous system, central nervous system, and specific brain regions (e.g., hippocampal gyrus, orbital cortex, lateral orbitofrontal cortex, and medial orbitofrontal cortex). These terms were used to extract directional relationships—specifically, upstream regulators and downstream targets—for COVID-19 and anxiety independently. Supporting references for each identified relationship are provided in **Supplementary Table 1** through 5.

Directional pathways were then constructed using two approaches: (1) from COVID-19 to anxiety by combining downstream targets of COVID-19 with upstream regulators of anxiety, and (2) from anxiety to COVID-19 by combining downstream targets of anxiety with upstream regulators of COVID-19.

Inclusion criteria for literature-derived relationships (edges) were as follows: each edge had to be supported by at least three independent literature sources, carry a signed polarity (positive or negative), and pass manual review to ensure that the supporting sentence(s) accurately described a biologically plausible association. In cases of conflicting evidence across sources, majority voting was applied to resolve discrepancies. These curated relationships formed the basis for constructing interpretable, biologically grounded, directional pathways linking COVID-19 and anxiety. For future pathway updates, we are now utilizing the ABT knowledge tool (https://www.gousinfo.com/cnabt/userguide.html), a licensed platform that enables expanded access to recently published biomedical literature.

**Figure 1:**
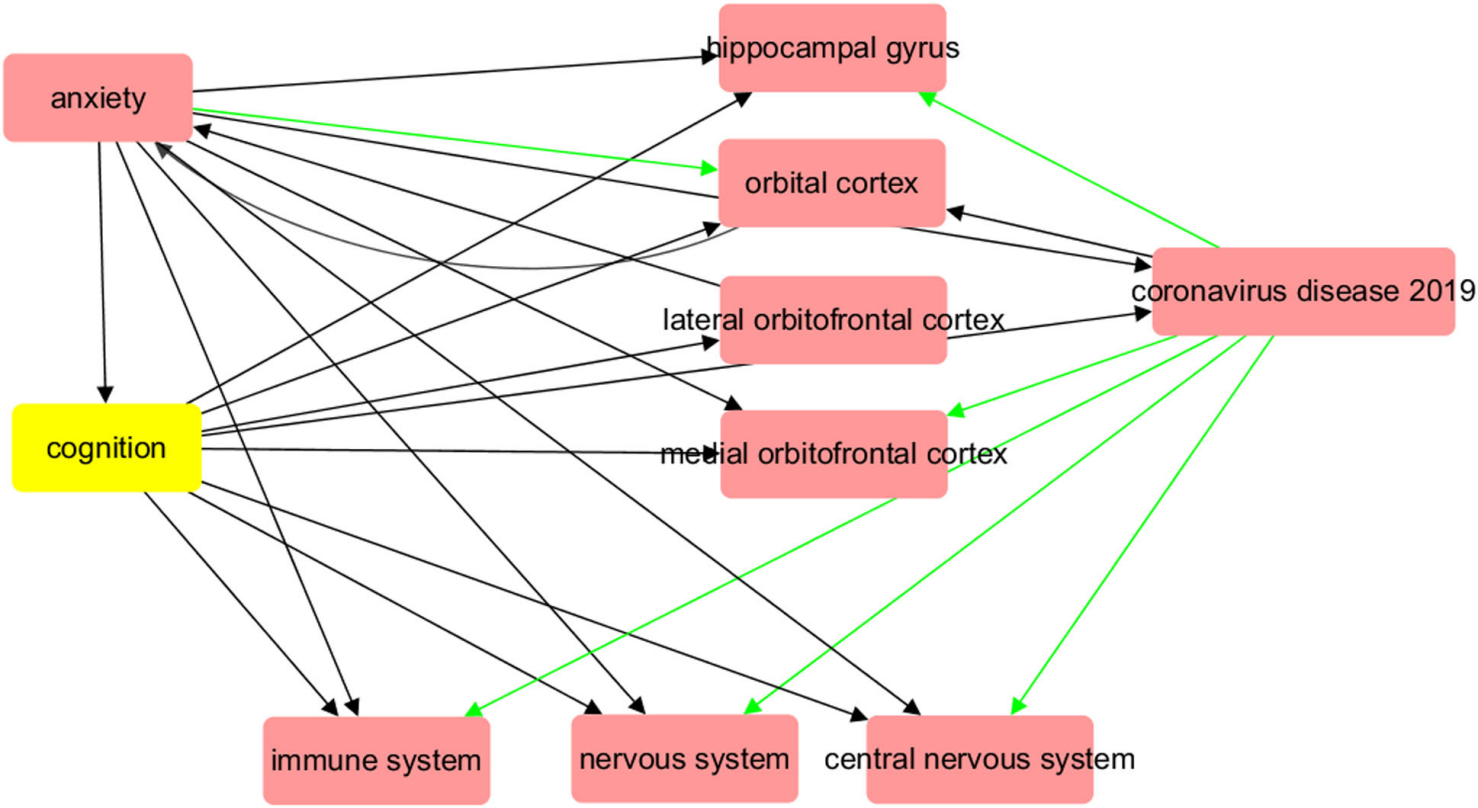
Biological abnormalities induced by COVID-19 at organ and system level may contribute to the development of major anxiety disorder. The red arrow symbolizes a positive impact, while the green arrow indicates a negative influence.

**Figure 2:**
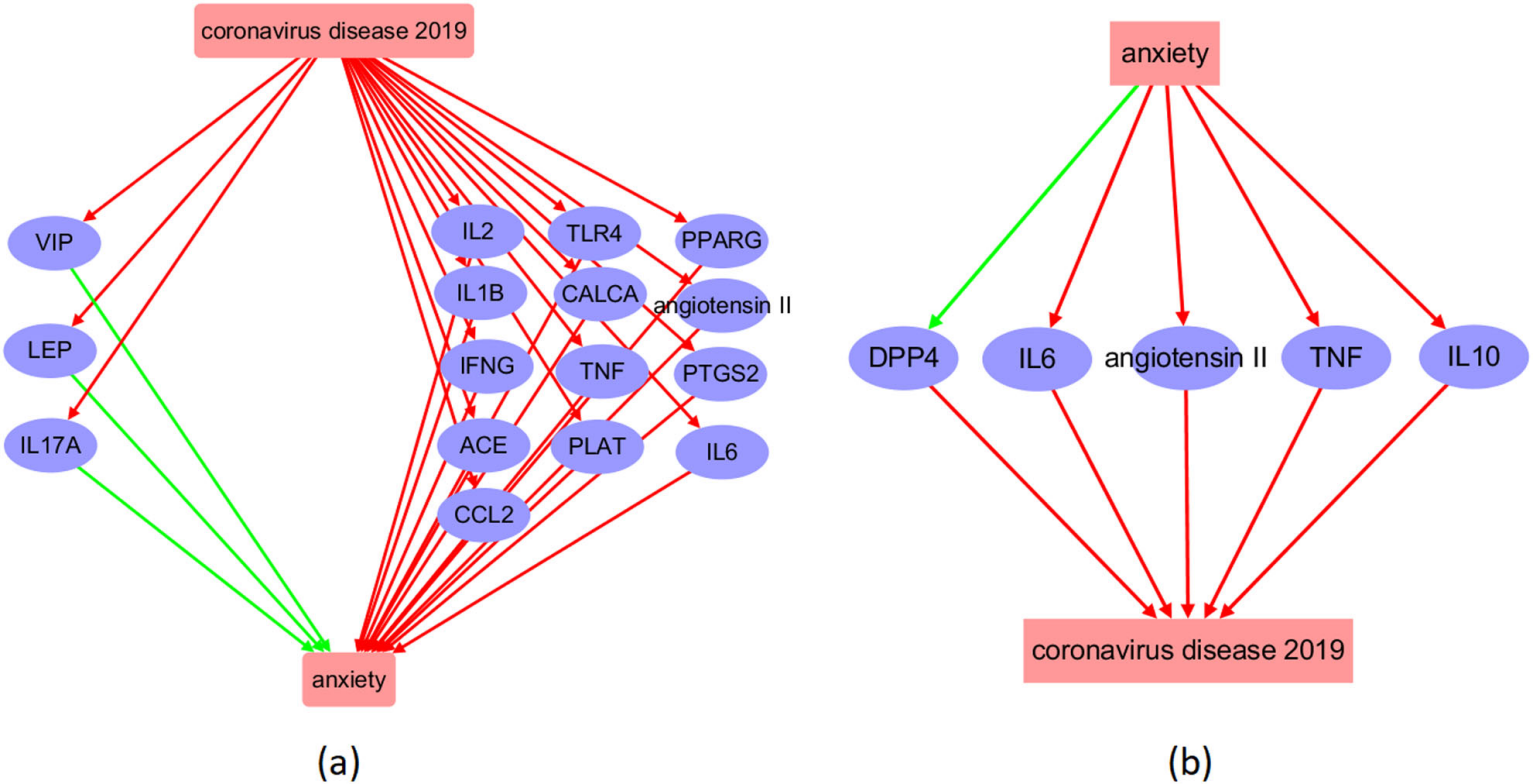
Molecular pathway connecting COVID-19 and anxiety. **(a)** COVID-19-driven pathway influencing anxiety; **(b)** Anxiety-driven pathway influencing COVID-19. The red arrow symbolizes a positive impact, while the green arrow indicates a negative influence.

While both directions of influence were explored during pathway reconstruction, only the COVID-19 → anxiety direction was evaluated with clinical and transcriptomic data in this study. The reverse direction, anxiety → COVID-19 severity (as illustrated in certain paths in **Figures 1**, **2b**), was reconstructed from systematically mined and manually validated literature relationships. These literature-based interactions reflect biologically plausible mechanisms—including immune modulation, HPA axis dysregulation, and neuroinflammatory priming—and are presented as hypothesis-generating for future empirical validation.

### 2.3 COVID-19 RNA expression data acquisition

To further investigate the molecules influenced by COVID-19 that affect anxiety, we performed a meta-analysis based on COVID-19 expression data. We obtained COVID-19 RNA array-expression datasets from GEO (https://www.ncbi.nlm.nih.gov/geo/) using the keyword “COVID-19”, resulting in 616 series datasets. To fulfill the study’s objectives, we applied the following criteria: 1) RNA expression by array was the data type; 2) The organism in the dataset was Homo sapiens; 3) The dataset conducted the COVID-19 patients vs. healthy control study design; and 4) The sample size was ≥10. We identified 8 datasets that met the selection criteria and used them for expression analysis, as shown in **Table 1**.

**Table 1:** The 8 COVID-19 RNA expression datasets from GEO.

Dataset GEOID	#Control	#Case	Country	Study Age	Sample Source	Sample Organism
GSE164805	5	5	China	3	PBMC	Homo sapiens
GSE164805	5	5	China	3	PBMC	Homo sapiens
GSE177477	18	18	Pakistan	3	blood	Homo sapiens
GSE177477	18	18	Pakistan	3	blood	Homo sapiens
GSE180226	3	3	USA	2	Lung	Homo sapiens
GSE183071	36	36	Spain	3	Blood; nasal; saliva	Homo sapiens
GSE213313	11	11	Norway	1	whole blood	Homo sapiens
GSE211378	40	40	USA	2	whole blood	Homo sapiens

‘Study Age’ was defined as the current year- the year of data submission.

### 2.4 Meta-analysis models

Meta-analysis was performed on 16 candidate genes identified from the constructed COVID-19–driven pathway influencing anxiety. One gene was excluded from the analysis due to insufficient publicly available expression data, resulting in a final set of 15 genes. For each of these, log2-transformed fold changes (effect sizes) and corresponding standard errors were calculated directly from raw gene expression data obtained from the Gene Expression Omnibus (GEO), across multiple independent COVID-19 case-control studies. Sample sources included peripheral blood mononuclear cells (PBMCs), whole blood, and other clinically relevant tissues, as detailed in **Table 1**.

All data were processed uniformly across studies. No cross-platform normalization or batch correction was applied, in order to avoid the potential introduction of additional noise or bias. Instead, between-study variation was addressed through model selection based on heterogeneity metrics. Specifically, heterogeneity was quantified using the total variance statistic Q and its degrees of freedom df , from which the $I^{2}$ statistic was computed as:


$${\text{I}}^{2}=\frac{Q-df}{Q}\times 100\%$$

If Q≤df , $I^{2}$ was set to 0 and a fixed-effect model was applied. Otherwise, a random-effects model was used. The PValue_Q value from Cochran’s Q test reflects the probability that the total variance is attributable solely to within-study variation. In both model types, within-study variance (standard error of each effect size) was incorporated directly into the weighted meta-analysis to ensure study-level reliability was accounted for.

Meta-analysis was conducted following the framework described by Borenstein et al. (38). Although the analysis focused on a limited, hypothesis-driven gene set, we applied the Benjamini–Hochberg false discovery rate (FDR) correction to control for multiple testing. However, due to the small number of tests, we report the original unadjusted p-values in the main text, while both raw and FDR-adjusted values are provided in **Supplementary Table 6**. All analyses were conducted using Matlab (R2017a version).

### 2.5 Analysis of influential factors

We carried out a multiple linear regression (MLR) analysis to determine the potential impact of various factors, such as study date, country of origin, and sample size, on the gene expression in patients with COVID-19. We reported the P-values for each of these factors to assess their significance.

## 3 Results

### 3.1 Zung Self-Rating Depression Scale

Among the 36 individuals who were asymptomatically infected with COVID-19, mild anxiety levels (AIS: 45~58) were observed in 9 patients, representing 25% of the sample. No severe or moderate anxiety was reported. **Table 2** provides the basic clinical characteristics of the patients assessed. Our findings are in agreement with previous research, suggesting that anxiety levels may be elevated even in asymptomatic COVID-19 cases.

**Table 2:** Sample information summary.

Attributes	Value
# sample	36
Gender	Female/Male: (17/19)
Age (year)	39.11 ± 12.18
SDS score (total)	44.62 ± 10.74
COVID-19 diagnosis	asymptomatic infection

Our study conducted a correlation analysis between age, gender, and anxiety level (AIS score) among asymptomatic COVID-19 patients. The results showed a mild inverse correlation between age and AIS score (Pearson r = –0.22, p = 0.19), suggesting that younger patients may be more susceptible to anxiety. No significant correlation was found between AIS and gender (r = –0.024, p = 0.87). However, a higher proportion of female patients was observed in the mild anxiety group. Although not statistically significant, this trend aligns with prior reports suggesting that females may experience higher anxiety levels due to both biological factors (e.g., hormonal fluctuations, HPA axis sensitivity) and sociocultural influences (e.g., caregiving roles, stigma, and exposure to stressors). Further research with larger samples is needed to confirm these patterns.

### 3.2 COVID-19 influence multiple organs connected with anxiety disorder

A functional pathway linking COVID-19 with brain regions (**Figure 1**) was reconstructed by summarizing the results of previous imaging studies, which included analyses of both structural and functional MRI data. **Supplementary Table 5** provides a catalog of references with PMID numbers that corroborate the connections depicted in **Figure 1**. As demonstrated in the illustration, COVID-19 has exhibited the capacity to harm numerous sections of the brain, such as diminishing grey matter thickness and tissue differentiation in the orbitofrontal cortex and parahippocampal gyrus. This damage extends to areas interlinked with the primary olfactory cortex, ultimately leading to a reduction in overall brain size. Many of these brain abnormalities are also pathological changes observed in patients with anxiety disorders, and they may be related to microvessel dysfunction in the brain caused by COVID-19. COVID-19 can also cause damage to the nervous system, central nervous system, and immune system, which are pathological features of anxiety disorders. Furthermore, all of these COVID-19-driven organ dysfunctions are linked to decreased cognition, which is an important feature of anxiety disorders. The pathway depicted in **Figure 1** may provide a possible association between COVID-19 and anxiety and an underlying mechanism.

### 3.3 Influence of COVID-19 on molecular promoters of anxiety

By conducting a literature search, we were able to identify 524 genes that were downstream targets of COVID-19, as evidenced by over 2,900 references. In addition, we identified 248 upstream regulators of COVID-19, which were supported by over 1,900 references. Furthermore, we found 199 downstream targets and 359 upstream regulators for anxiety, supported by over 491 and 4,800 references, respectively. Please refer to **Supplementary Table 1** through **Supplementary Table 4** for comprehensive information about the discovered supporting sources. This includes details such as PMID, publication year, DOI, and the nature of the relationship’s polarity. Based on this information, we constructed the COVID-19-driven molecular pathways depicted in **Figure 2a**, and the anxiety-driven molecular pathways shown in **Figure 2b**.

The molecular pathways illustrated in **Figure 2a** demonstrate that COVID-19 has the potential to activate 16 upstream regulators of anxiety, with 13 of them (81.25%) acting as anxiety promoters and the remaining 3 (18.75%) serving as inhibitors. Conversely, anxiety may impact the function of five COVID-19 regulators, including the activation of four proteins (IL6, angiotensin 2, TNF, and IL10) and the inhibition of one protein (DPP4). These pathways suggest a reciprocal and detrimental relationship between COVID-19 and anxiety at molecular level.

### 3.4 Filter using meta-analysis

**Figure 3:**
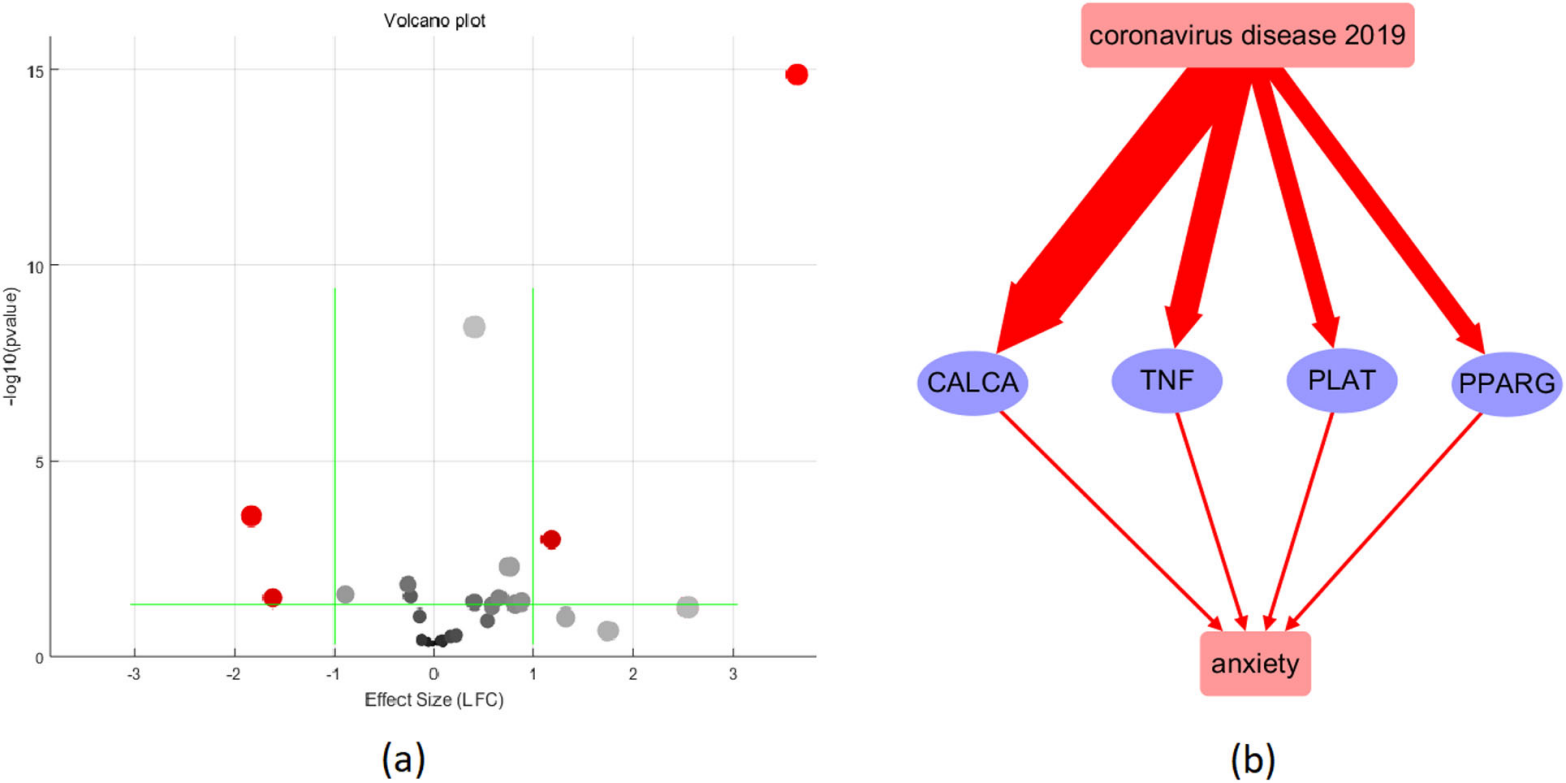
Validation of the 16 COVID-19-driven anxiety regulators using meta-analysis. **(a)** Volcano plot of the meta-analysis results; **(b)** pathway after validation: red color represents activation; the wider the line, the higher the expression level.

To confirm the relevance of the COVID-19-associated molecules in the pathway depicted in **Figure 2a**, we performed a meta-analysis using eight COVID-19 RNA expression datasets. After removing those proteins that displayed contradictory or insignificant expression changes, only four proteins remained: CALCA, TNF, PLAT, and PPARG. These are presented in **Table 3** and **Figure 3**.

**Table 3:** Meta-analysis and MLR-analysis results of the 4 proteins in **Figure 3b**.

Gene Symbol	meta-analysis results				MLR analysis results (p-value)		
	Using Random Effects Model?	#Study	Effect Size	p-value	Country	#Sample	Study Age
CALCA	1	3	3.64	1.33E-15	0.50	1	1
TNF	1	6	1.28	5.36E-4	0.0026	0.98	0.91
PLAT	0	3	0.41	3.77E-9	0.50	1	1
PPARG	0	3	0.40	0.041	0.50	2.22E-16	2.22E-16

The meta-analysis results showed that CALCA had a significant effect size of 3.64 with a very low p-value of 1.33E-15, indicating a strong association with COVID-19. TNF also had a significant effect size of 1.28 with a low p-value of 5.36E-04. PLAT had an effect size of 0.41 with a very low p-value of 3.77E-09. PPARG had a significant effect size of 0.4 with a p-value of 0.041. Overall, these results suggest that CALCA, TNF, and PLAT are strongly associated with COVID-19, while PPARG has a relatively weaker association.

**Table 3** also presents the results of the MLR analysis conducted to examine the effects of sample size, data collection date (Study Age), and sample region (Country) on the expression levels of COVID-19-driven molecules in **Figure 3**. The analysis showed that sample size and data collection date have a significant impact on the expression levels of PPARG, whereas sample region is a risk factor for the expression of TNF. However, there was no significant impact of these factors on the expression levels of the other genes tested.

## 4 Discussion

Anxiety is highly prevalent in COVID-19 patients, which contributes to the burden of the pandemic (13, 14, 39). Despite increased public interest in exploring the possible association between the two conditions (15), limited studies have investigated the pathophysiological mechanism underlying the high comorbidity of COVID-19 and anxiety disorders (40). In this study, we conducted a Zung SAS analysis to validate the anxiety status in COVID-19 patients and reconstructed functional and molecular pathways to explore the possible mechanism connecting COVID-19 and anxiety. Additionally, we performed a meta-analysis based on eight COVID-19 datasets to further validate literature-based COVID-19-driven proteins. Our findings revealed multiple molecule pathways indicating a mutual vicious effect between COVID-19 and anxiety disorders (39, 40).

In our Zung SAS analysis, it was observed that approximately a quarter of COVID-19 patients experienced mild symptoms of anxiety, which is consistent with previous research (39, 41). Notably, our study was conducted in April 2022 on patients who had been infected with the Omicron BA.2 variant strain of the coronavirus and were asymptomatic. Our findings suggest that COVID-19 can lead to increased anxiety even in those who are asymptomatic. Additionally, our study indicated that younger COVID-19 patients were more likely to experience anxiety, which is in line with previous studies (42). Nonetheless, there was a similar effect on both male and female patients, despite 66.7% (six out of nine) of patients with anxiety status being female, which may be attributed to the relatively small sample size of our study.

The functional pathways we composed, as shown in **Figure 1**, indicated that COVID-19 may affect the structure and function of several brain regions, such as the hippocampal gyrus, orbitofrontal cortex, and olfactory cortex, which could lead to dysfunctions in the immune system, nervous system, and microvessels (16–21). These pathological changes have also been associated with anxiety in previous studies (22–24), and interestingly, they are frequently linked to decreased cognition, which is a hallmark of anxiety (43, 44). These pathological changes may represent a shared pathophysiological mechanism between COVID-19 and anxiety at the system and organ levels, with decreased cognition being a key factor linking COVID-19 with increased anxiety prevalence.

The reconstructed molecular pathway (**Figure 2**) reveals that COVID-19 could activate 13 anxiety promoters and three anxiety inhibitors, indicating a majority of negative effect on anxiety. Further validation using expression meta-analysis show that the influence of COVID-19 on anxiety may be more likely through the upregulation of four anxiety promoters, including CALCA, TNF, PLAT, and PPARG, as shown in **Figure 3b**. Specifically, while our clinical cohort comprised patients infected with the Omicron variant (BA.2), these data were used primarily as supplementary evidence to support prior research establishing a link between COVID-19 and anxiety-related outcomes (References 11, 12). The goal of our bioinformatics analyses was to uncover generalized molecular mechanisms associated with COVID-19–related anxiety, rather than variant-specific effects. Therefore, while the lack of Omicron-specific samples in the public datasets is a limitation, it does not undermine the broader interpretability of our pathway reconstruction and meta-analysis findings.

CALCA, which encodes the protein calcitonin gene-related peptide (CGRP), is positively associated with COVID-19. Several studies suggest that levels of CGRP and procalcitonin are elevated in COVID-19 patients (45–47). Moreover, higher levels of procalcitonin, ferritin, CRP, lactate dehydrogenase, interleukin-6, and D-dimer are linked to poor prognosis and high mortality risk for COVID-19 patients (47–51). CALCA has a positive regulatory influence on anxiety. CGRP injection caused significant anxiety and pain responses in female mice (52). CGRP neuropeptides affect stress responses and anxiety in vertebrates, which strongly influence sleep (53). Infusion of CGRP into the bed nucleus of the stria terminalis was reported to potentiate anxiety while activating bed nucleus of the stria terminalis targets (54). CGRP within the bed nucleus of the stria terminalis interacts closely with corticotropin-releasing factor signaling to induce anxiety and mediates behavioral stress responses (55). Infusions of CGRP within the bed nucleus of the stria terminalis increase anxiety measures in the plus maze, while infusions of a CGRP antagonist decrease sustained startle increases produced by the predator odor 2,5-dihydro-2,4,5-trimethyl-thiazoline (56). Additionally, the neuropeptide Y-CGRP ratio may play a role in anxiety and in the action of antipsychotic drugs (57). These evidence establish the COVID-19→CALCA→anxiety pathway.

Increased levels of TNFα were observed in patients with coronavirus disease 2019 (COVID-19) compared to controls and these levels were associated with severity and poor survival (58, 59). Administration of TNF-α increases anxiety, anhedonia, and depressive feelings in humans and rodents (60, 61). Inflammatory cytokines, including TNF-α, are involved in the development of anxiety and depression by triggering neuronal damage and microglial dysfunction (62–64). Additionally, prenatal discrimination is associated with postnatal anxiety and depression mediated by TNF-α methylation (65). Variations in TNF-α are also associated with state anxiety in oncologic patients and their family caregivers (66). These evidences establish the COVID-19→TNFα→anxiety pathway.

The enzyme tissue plasminogen activator (PLAT) is involved in various brain functions involving behavior and emotions, including fear/anxiety in the amygdala and plays a crucial role in regulating anxiety and fear responses in the amygdala, as evidenced by several studies (67, 68). Acute stress activates structural plasticity and enhances anxiety in the amygdala through PLAT (69). PLAT mediates stress-induced anxiety by promoting neuronal activity in the medial amygdala and the rapid outgrowth of presynaptic connections (70, 71). Conditional deletion of PLAT in the central amygdala leads to locomotor hyperactivity and reduced anxiety (68). It has been reported elevated levels of PLAT in COVID-19 infections (72–74). These evidence establish the COVID-19→PLAT→anxiety pathway.

PPARG is a nuclear receptor that plays a crucial role in regulating glucose and lipid metabolism. A study revealed the importance of PPARG expression in B cells during the primary and secondary immune response, indicating that the PPARG activation pathway can be used to enhance the humoral immune response (75). Severe acute respiratory syndrome coronavirus 2 infection has been found to upregulate PPARG expression and other key lipid metabolic enzymes *in vitro* (76). Apart from metabolic effects, PPARG has been implicated in anxiety and depression regulation. Dysregulated PPARG activity and impaired MME and ACE peptidase expression in the amygdala have been suggested as a possible mechanism leading to pathological anxiety development, with CCK-4 accumulation in the brain being a crucial link (77). Furthermore, the genetic deletion of neuronal PPARG has been found to reduce stress-induced anxiety and alleviate the expression of somatic and affective nicotine withdrawal symptoms in animal models (78). Together, these findings establish the COVID-19-induced PPARG-anxiety pathway.

It’s interesting that three out of the four genes discussed above, namely PLAT, TNF, and PPARG, have been implicated with a possible connection to cognition. The gene PLAT, responsible for encoding tissue-type plasminogen activator (tPA), is influenced by ethanol to increase in astrocytes and brain regions like the cortex and hippocampus [PMID: 29885422]. This upsurge, observed in fetal alcohol spectrum disorders (FASD), suggests a potential involvement in cognitive impairment, deficits, and mental decline due to modified tPA levels affecting neuronal adaptability as a response to prenatal alcohol exposure. The relationship between TNF and cognition is complex and varied. Research proposes that mitigating the elevation of TNF-α through strategies such as dexmedetomidine [PMID: 37550504], inhibiting TNF-α release via the mmu_circ_0001442/miR-125a-3p/NUFIP2 pathway [PMID: 37550899], decreasing TNF-α expression using probiotics that curb gut inflammation [PMID: 37571319], and reducing TNF-α alongside other inflammatory markers [PMID: 37562566] can alleviate cognitive impairments. Conversely, heightened TNF-α levels have been correlated with cognitive deterioration, evident in AD mice with Gpr34 knockdown [PMID: 37557947] and in Alzheimer’s-related hypothalamic inflammation [PMID: 37559092]. These findings underscore the intricate interplay between TNF and cognition, suggesting its potential as a target for interventions aimed at cognitive enhancement.

Conversely, the PPARG gene plays a multifaceted role in cognition. It interacts with genes and miRNAs implicated in neuroinflammation and neurodegeneration, contributing to amnestic mild cognitive impairment (aMCI) and sporadic Alzheimer’s disease (AD) [PMID: 35250545]. High-intensity interval training (HIIT) can avert cognitive deficits by influencing PPARG gene expression, potentially forestalling cognitive impairment [PMID: 36503461]. The Pro12Ala polymorphism of PPARG is tied to cognitive decline, particularly among black males, suggesting a situation-dependent connection [PMID: 29116943]. Additionally, PPARG has been linked to cognitive decay and mental deterioration in AD and postoperative neurocognitive disorder [PMID: 33920138, 32412807]. PPARG’s involvement in cognitive impairment and its potential as a therapeutic target necessitate further investigation. Taken together, these findings establish that the alterations in PPARG and TNF expression prompted by COVID-19 may also contribute to the decline in cognitive function associated with anxiety.

In addition, according to a literature-based pathway, anxiety may upregulate multiple cytokines (IL6, IL10, and TNF) and angiotensin II, which are associated with harmful effects during COVID-19. Specifically, the upregulation of angiotensin II has been shown to increase pulmonary vascular leakage, which can cause lung injury during COVID-19 infection (79). Moreover, the cytokines TNF, IL5, and IL10 have crucial roles in COVID-19 prognosis (80, 81). However, anxiety has also been found to inhibit DPP4 levels (82). It has been suggested that the inhibition of DPP4 could decrease COVID-19 severity by reducing inflammation and enhancing tissue repair beyond glucose lowering (83). Experiment data are needed to validate the anxiety-driven molecular pathway influencing COVID-19.

It should be noted that, while the downstream effects of COVID-19 on anxiety were directly supported by clinical and transcriptomic data in this study, the reverse pathway—from anxiety to COVID-19 severity—was reconstructed from systematically mined and manually validated literature relationships, and should be interpreted as hypothesis-generating rather than conclusive. Moreover, this study is primarily correlational, causal inferences cannot be drawn from our clinical or molecular findings. In future work, approaches such as Mendelian randomization—leveraging genetic variants as instrumental variables—may help assess potential causal links between COVID-19 and anxiety. Additionally, longitudinal cohort studies could provide valuable insight into the temporal and directional dynamics of these relationships, especially as large-scale GWAS and omics data become increasingly available.

This study has several limitations. First, the modest sample size (N = 36), while sufficient for detecting group differences in Anxiety Index Scores (AIS) with 95% power, limits generalizability and precludes full psychometric validation of the Zung Self-Rating Anxiety Scale (SAS), which typically requires N ≥ 300. The small anxiety subgroup (n = 9) also restricted stratified analyses; thus, we limited comparisons to a binary anxiety vs. non-anxiety classification. Second, COVID-19 vaccination status was not collected, limiting our ability to account for vaccine-related biological or psychological effects. Although recruitment occurred in April 2022—during the post-vaccine period—vaccination data were not routinely integrated into anxiety-focused COVID-19 studies at that time. Third, while our literature-based pathway reconstruction and transcriptomic meta-analysis provide molecular insight, they are correlative and reflect the state of published evidence as of May 2023. Future studies should incorporate Mendelian randomization approaches to assess causal directionality and update pathway findings using continuously refreshed biomedical databases and tools. Fourth, while the identification of candidate mediators (e.g., TNF, CALCA, PPARG, PLAT) is supported by literature evidence and transcriptomic meta-analysis, the molecular findings remain hypothesis-generating. These results complement the clinical SAS data by proposing mechanistic underpinnings for the observed anxiety symptoms in COVID-19 patients. Future studies integrating proteomic, immunological, or cellular validation will be valuable to experimentally verify the molecular pathways proposed here.

## 5 Conclusion

Our integrative analysis provides evidence that COVID-19 and anxiety engage in a bidirectional, deleterious interplay at the molecular level. Specifically, SARS-CoV-2 infection appears to upregulate multiple pro-inflammatory cytokines and secreted proteins (e.g., CALCA, TNF, PLAT, PPARG), which are known to promote anxiety phenotypes, while anxiety itself may modulate key regulators of viral pathophysiology. Moreover, we highlight decreased cognitive function—stemming from both neuroinflammatory damage and dysfunctional molecular pathways—as a potential mediator linking COVID-19 with heightened anxiety prevalence.

## Data Availability

The raw data supporting the conclusions of this article will be made available by the authors, without undue reservation.
